# Female cardioprotection in ischemia/reperfusion: Isn't a SUR thing anymore?^☆^

**DOI:** 10.1016/j.jmccpl.2024.100089

**Published:** 2024-08-13

**Authors:** Annalara G. Fischer, Helen E. Collins

**Affiliations:** Center for Cardiometabolic Science, Christina Lee Brown Envirome Institute, Division of Environmental Medicine, Department of Medicine, University of Louisville, Louisville, KY, USA

Without prompt reperfusion following a myocardial infarction (MI), an ischemic insult will culminate in irreversible damage, provoking overwhelming cardiomyocyte death and the eventual formation of a non-contractile scar. While reperfusion ultimately improves clinical outcomes by reducing mortality and preserving left ventricular function, adverse consequences may manifest in a phenomenon termed ischemia-reperfusion (I/R) injury. Despite the benefit that reperfusion affords, complications of I/R injury may arise, including myocardial stunning, lethal reperfusion injury, or no-reflow phenomenon [1]. I/R injury is characterized by the generation of reactive oxygen species, mitochondrial and metabolic dysfunction, inflammation, and the dysregulation of ion channel homeostasis (e.g., Ca^2+^, K^+^, Na^+^, etc.) [1]. Although Ca^2+^ overload is thought to be a major contributory factor in I/R injury and the subsequent activation of cell death pathways, loss of the ATP-sensitive K^+^ (K_ATP_) channel current has also been implicated. Indeed, pharmacologic agents that open K_ATP_ channels, such as nicorandil and pinacidil, have protective effects in the setting of ischemic injury, including a reduction in infarct size [2]. The protection afforded by K_ATP_ channel openers have been likened to the protection seen during ischemic preconditioning, suggesting that the opening of K_ATP_ channels may play a key role in this type of cardioprotection. Despite this extensive knowledge regarding the mechanisms underlying I/R injury, many of these seminal studies only examined the consequences of I/R injury and the underlying contributions of the K_ATP_ channel in males, with little known about whether this is also the case in females.

Significant differences exist between females and males following myocardial ischemic injury. Preclinical models reliably indicate premenopausal female animals have increased survival, less adverse ventricular remodeling, and preserved cardiac function versus age-matched males in response to ischemic injury [3]. However, following menopause, studies suggest that females have comparable and sometimes greater levels of injury in response to ischemia, suggesting that sex hormones could play a significant role in the protection of premenopausal females. The premenopausal protection seen in females has resulted in historical reductions in their inclusion in studies focused on ischemic injury because, being essentially protected from injury, it is hard to study the underlying mechanisms that contribute. Issues such as these have contributed to a paucity of knowledge regarding how females respond to cardiac injury. Despite this, recent studies suggest that K_ATP_ channels may be a critical regulator of post-ischemic responses in females [4], highlighting a potential exploitable sex-specific target for novel therapeutic strategies.

The findings of the study by Wexler et al. provide insights into the role of SUR2A-55 in the female heart during ischemic injury. SUR2A-55 is a splice variant of the SUR2A K_ATP_ channel subunit, which has been shown to accumulate with reductions in SUR2A expression [5]. Previous studies by this group have noted that overexpression of SUR2A-55 was protective in males in response to I/R injury [5]. Hence, the goal of the present study was to examine whether this protection also occurs in females, given that sex-specific changes have already been observed with SUR2A, K_ATP_ channel expression, and responses to K_ATP_ channel openers [6]. The findings show that female mice with SUR2A-55 overexpression (SUR2A-55 OE) exhibit increased I/R injury versus wild-type mice, including indications of adverse ventricular remodeling. It is important to note that the studies were conducted without sham-operated controls, so baseline deficits in cardiac structure or function in SUR2A-55 OE mice cannot be ruled out. Nevertheless, the exacerbated I/R injury in SUR2A-55 OE mice was associated with transcriptional changes in several key mitochondrial genes associated with oxidative metabolism, increased mitochondrial membrane potential, and reduced sensitivity to both ATP and diazoxide, the opposite of what was previously documented in male mice [5]. Although these findings are exciting, the underlying mechanisms contributing to this differential response between the sexes have yet to be elucidated.

Although this study provides new information on how the female heart adapts to cardiac stress imposed by I/R injury, several questions remain unanswered. Remaining unclear are the fundamental mechanisms of how differential SUR2 splicing occurs in females versus males and whether this could be targeted directly. It would be also be important to elucidate the direct impact of SUR2A-55 OE on K_ATP_ channel activity. As the authors mention, it is important to identify not only the regulatory binding equivalent of Kir to SUR2 in mitochondrial K_ATP_ channels but to also tease apart the contribution of mitochondrial versus sarcolemmal K_ATP_ channels, given that they both express SUR2 and could be sensitive to the splice variant. Moreover, additional studies are warranted to examine SUR2A-55 levels in the context of I/R injury in wild-type mice. For example, knowledge of when changes in expression of the splice variant occur during I/R could provide more solid mechanistic footing and yield information applicable to temporal targeting SUR2A-55. Determining whether the detrimental ischemic response in female SUR2A-55 OE mice could be reversed with K_ATP_ channel inhibition (e.g., with glibenclamide) could also prove useful in teasing out additional mechanistic information.

The findings of Wexler et al. are unique because few studies show worsened outcomes in female mice with I/R injury. Although the combination of SUR2A-55 OE with ischemic injury appears sufficient to overcome the protective effects of estrogen, additional studies are needed to determine the impact of SUR2A-55 in conditions comparable to menopause, when females are truly vulnerable to ischemic injury. Targeting the K_ATP_ channel may also yield additional insights into at-risk female populations, such as postmenopausal diabetic females, who have an elevated risk of myocardial ischemia and who may be taking K_ATP_ channel therapeutics. In addition, this study highlights the fact that we need to have a greater appreciation for the presence of channel splice variants, such as SUR2A-55, and the key roles that they may play in shaping responses to pathological stressors.

One notable limitation of the study is that it relies on inferences made from historical data obtained in males to yield information on sex-dependent changes in I/R injury. Although the Sex and Gender Equity in Research guidelines encourage that male and female data are analyzed and presented separately to report sex differences accurately, this description is used for data collected during the same experimental series. Reliance on historical data could result in an under- or over-estimation of findings and does not rule out batch effects between time-separated interventions. If the inferred sex-dependent changes hold, additional examination would be required to tease apart the specific hormonal and perhaps also chromosomal contributions to changes in K_ATP_ regulation and function between the sexes. Despite the fact that much work is left to do, the study from Wexler et al. has invariably set the stage for additional investigations that will tease apart the contributions of the K_ATP_ channel to the differential sex-dependent responses observed in I/R injury, which could lead to future therapeutic strategies aimed at targeting the KATP channel and relevant splice variants (Fig. 1).

**Fig. 1:**
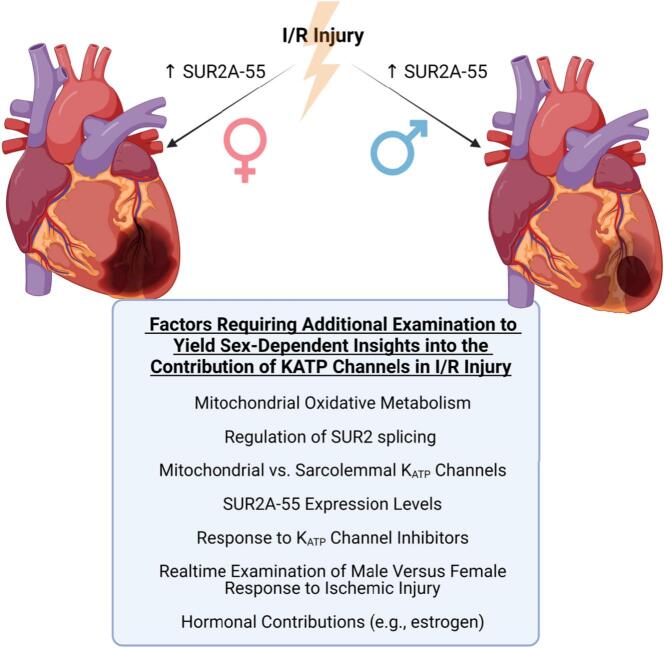
Schematic of the additional considerations needed to interrogate the importance of the mitochondrial K_ATP_ channel in sex-dependent responses to ischemic injury. This Figure highlights key findings from the present study of Wexler et al., whereby females with overexpression of the K_ATP_ channel splice variant, SUR2A-55, exhibited exacerbated injury in response to ischemia and reperfusion (I/R). The Figure also highlights the historical findings from the same group in male SUR2A-55 overexpressing mice showing protection from I/R injury. Key factors that need further investigation regarding the contribution of the SUR2A-55 splice variant to sex-dependent responses to ischemic injury are also noted. This Figure was generated using BioRender software.

## CRediT authorship contribution statement

**Annalara G. Fischer:** Writing – original draft, Writing – review & editing. **Helen E. Collins:** Conceptualization, Funding acquisition, Resources, Writing – original draft, Writing – review & editing.

## Declaration of competing interest

The authors declare the following financial interests/personal relationships which may be considered as potential competing interests: Helen E. Collins reports financial support was provided by University of Louisville. Helen E. Collins reports a relationship with NIH NHLBI Grant that includes: funding grants. If there are other authors, they declare that they have no known competing financial interests or personal relationships that could have appeared to influence the work reported in this paper.
